# Enhancing community engagement, public involvement, and social capital through researchers’ participation in community dance projects: unexpected outcomes in underserved communities

**DOI:** 10.1186/s40900-024-00616-9

**Published:** 2024-08-02

**Authors:** Rebecca Pritchard, Natalie Darko, Elizabeth Stevenson

**Affiliations:** 1 BMTO, Old Medical School, University of Edinburgh, Teviot Place, Edinburgh, EH8 9AG UK; 2 NIHR Leicester BRC, Leicester Diabetes Centre, Leicester General Hospital, University of Leicester, Gwendolen Road, Leicester, LE5 4PW UK

**Keywords:** Community engagement, Social capital, Public involvement, Underserved communities, Ethnic minority, Dance

## Abstract

**Background:**

The Dance and Health project aimed to promote public involvement in health research. Public involvement leads worked with project partner community groups, Aakash Odedra Dance Company and Moving Together, to develop a community engagement project with people living in low-socioeconomic areas/deprivation and diverse ethnic minority groups. Dance and Health included a weekly 60-min dance class and 30 min of facilitated health science discussion, that could either be a public involvement discussion for a research project, an activity about a particular biomedical research theme or ongoing discussions with a visiting researcher. The goal of this paper is to explore the impact of the Dance and Health project on the social capital of participants and provide key learnings on how to engage and build partnerships with people from underserved groups in health research contexts.

**Methods:**

Qualitative interviews and focus groups were completed which explored participant and dance tutor experiences in community venues. Participants were aged between 22 and 90, most were female and were from Asian ethnic minority groups and White British groups living in deprived neighbourhoods in Leicester. Qualitative data were analysed using qualitative content analysis.

**Results:**

The responses to the Dance and Health project were positive across all the focus groups. Central themes identified were *Feedback on the Project, Motivation to Exercise, Criticism of the NHS, Mental Wellbeing, Engagement in a Post Pandemic New Normal, Accessibility and Inclusivity, Empowerment and Building Social Capital.*

**Discussion:**

The focus groups evidenced that the project had broad impact. Participants expressed empowerment and ownership and described a range of social capital enrichment generated through the project including networks and friendships, access to the institutional resource of health science, and the opportunity to engage with a health and leisure activity that was valued and meaningful.

**Supplementary Information:**

The online version contains supplementary material available at 10.1186/s40900-024-00616-9.

## Abstract (plain language summary)

A research team worked with community dance schools in Leicester to try to get more people from the Asian community and from poorer neighbourhoods involved in research and science. By taking part in the project, people benefitted from an opportunity to exercise and to learn about and influence health research taking place locally. However, the researchers also noticed that there were other benefits to the project. Dancing and learning about health science helped people bond and form new friendships. The researchers joined in, and this helped build friendships and trust between participants and people working in science and research. This is called bridging which is where we bring people together from different groups. The dance sessions made the researchers more approachable and less intimidating, so it was easier to trust them and build friendships. Bridging and bonding are ways of building *social capital*. Social capital describes the social resources that people can access in their neighbourhoods and communities e.g. facilities (village halls or clubs) and support networks. Researchers delivered focus groups in which participants talked about *Feedback on the Project, Motivation to Exercise, Criticism of the NHS, Mental Wellbeing, Engagement in a Post Pandemic New Normal, Accessibility and Inclusivity, Empowerment and Social Capital.* This supported the idea that projects where we try to engage with communities can build social capital. This is most likely when projects provide safety and accessibility, (1) and flexibility, a sense of belonging, commitment, communication, being genuine, relevance, sustainability (4).

**Supplementary Information:**

The online version contains supplementary material available at 10.1186/s40900-024-00616-9.

## Background

This paper seeks to share good practice related to the Dance and Health project, challenge policy makers and infrastructure managers to consider how they measure and maximise impact, and reflect on the alignment of macro and meso objectives of the National Institute for Health and Care Research (NIHR), infrastructure hosts such as universities and hospital trusts, Biomedical Research Centres and other NIHR infrastructure and the Department of Health.

Community engagement has positive impacts in terms of health behaviours, health outcomes, and feelings of self-efficacy which are in turn related to successful adoption of positive health behaviours, and the perception of social support, and specifically in excluded communities [1]. It represents a promising but under resourced approach to public health and health sciences engagement and involvement. Few studies have examined the effect of community engagement on social and human capital, but their findings suggest that community engagement is a way to develop resources within communities [1]. Arts based (health science) engagement in particular offers a powerful tool for community engagement with the potential to impact positively on health inequalities through the process of building social capital [2]. Arts projects can build social cohesion, which is related to better health, yielding both personal and community benefits [3]. With health behaviour embedded in cultural and community norms, collective creativity presents an opportunity to build social cohesion with a focus on increasing healthy choices and encouraging health behaviours [3]. Lower levels of health literacy and English literacy amongst migrant populations, including a specific lack of understanding of type 2 diabetes [4], a lack of knowledge about health services and related lower use of health services, poor compliance with medicines (possibly related to use of alternative therapies) and poor adherence to self-management (possibly related to the ethnocentricity of existing self-management programmes) [5] may contribute to risk [6] and therefore underpin health inequalities. O’Mara-Eves [1] asserts that, to be effective, community engagement projects need to be safe and accessible (in the broadest sense). Successful community engagement requires flexibility, a sense of belonging, commitment communication, being genuine, relevance, and sustainability [7].

Bollywood, Zumba and Garba, are social and cultural types of dancing where people move and laugh and learn together. They have potential to offer a range of social and emotional benefits. These benefits include social interaction and emotional rewards [8], social engagement [9] and even a space in which people can negotiate their understanding and experience of ageing [10]. These benefits are of course in addition to physical benefits [11] like postural stability, physical performance [12] better balance, and coordination [9]. Thus dance and health science is a unique art-science interface offering the power to attract an audience [13], reduce social isolation [8, 9] and therefore, impact social capital and health inequalities [1]. In a project with an over-arching focus on lifestyle diseases, dance also offers an opportunity to increase physical activity; our choice of art offers synergy with our topic of discussion.

Public involvement in health research emphasises the inclusion of diverse patient experiences and insights to enrich study design, implementation, and outcomes. This approach aligns with Pierre Bourdieu's concept of social capital, which he describes as the actual and potential resources linked to the possession of a durable network of more or less institutionalised relationships of mutual acquaintance and recognition [14]. In the context of public involvement and community engagement, social capital can be fostered through both bonding and bridging mechanisms. Bonding refers to the strengthening of relationships within an homogenous group, enhancing mutual support and shared understandings. Bridging, on the other hand, extends beyond similar groups to connect disparate groups, facilitating broader perspectives and resource sharing [15]. This dual approach is critical in health research, as it enables underserved groups to gain visibility and voice, thereby potentially enhancing their social capital. Through strategic public involvement, health research can not only address but also harness the strengths of diverse communities, leading to more equitable health outcomes and a more inclusive research process.

Building on the foundational understanding of social capital in public and patient involvement, it becomes evident that community engagement not only fosters relationships and networks but also facilitates empowerment and social mobilisation towards greater distributive justice. The enhancement of social capital through bonding and bridging, as discussed earlier, aligns with the findings of Ocloo et al. [16] and Cacari-Stone et al. [17], which suggest that empowered communities can actively influence social structures to promote equity. Moreover, the work of Sung [7] and O’Mara-Eves et al. [1] further supports the notion that by leveraging these networks and engaging communities, there is a tangible path towards addressing health inequalities. Roura [18] consolidates these viewpoints by asserting that community engagement can directly impact health disparities through the mechanisms of distributive and relational justice, thus linking increased social capital with tangible improvements in public health outcomes.

### Project aim

The overarching aim of the project is to explore the potential of a model of community engagement to deliver accessible and inclusive public involvement and engagement opportunities in the South Asian communities of Leicester and assess the impact of the model.

## Method

### Project background and setting

The Dance and Health project has been running since 2017. As a model of community engagement and associated principles and practice, it has expanded from a week long outreach intervention to a long-term project in three very different neighbourhoods, supported by a team of public involvement staff and community partner staff. It continues to support public involvement embedded in a model of community engagement that was iteratively developed between 2017 and the present, through a process of action research [19] incorporating structured whole team reflection and increasing coproduction. Logistically simple, comprising a weekly 60 min dance class and 30 min of facilitated health science discussion it is relationally complex. The project team, whilst holding particular skillsets, participated in all aspects of the project i.e. dance tutors joined in discussions and the researcher joined in the dancing.

Sessions are delivered in 3 blocks throughout the year, typically of 12–13 sessions. The discussion component may be public involvement for a research project, a facilitated discussion with an expert, an activity about a particular theme or ongoing discussions with a visiting researcher. Working effectively with the relation complexity has allowed the project to yield surprisingly broad impacts.

Initially intended as a model to achieve “out of the box” public involvement for the NIHR Leicester Biomedical Research Centre and Leicester Diabetes Centre in under-represented communities offering culturally attractive activity, the project developed into a transformative flagship project exploring health inequality and building social capital through multiple cycles of action research. In this article, the final cycle of action research, which incorporated a more comprehensive focus on impact assessment, is described.

The project is currently active in three Leicester neighbourhoods (and launching in a fourth), two of which, Belgrave and Braunstone, are included in this analysis. The third active site, Oadby Wigston, was not included owing to temporary staffing constraints. Leicester is the largest city in the East Midlands and is extremely diverse and very deprived. 43% of the population are Asian, and there are also notable Eastern European, Black African and Black Caribbean populations. 41% of the population were born outside the UK. Of the 317 local authorities in the UK, Leicester is the 32nd most deprived [20]. Belgrave has a population of 20,565 people, of which the majority (85.4%) identify as having Asian ethnicity and Hindu religion (71.8%) although there is also a notable Muslim population (12.5%). Belgrave is in the top 30% of most deprived neighbourhoods in the UK [21]. Bollywood dance was selected as a culturally appealing option in Belgrave. Braunstone (including Rowley Fields) has a population of 17,258, of which 70% identify as white, and 20% as Asian. 37% of the population are Christian, 9% Sikh and 8% Hindu. Braunstone is in the top 10% most deprived neighbourhoods in the UK [21]. In Braunstone, Zumba dance was used.

Whilst the project did not actively exclude anyone, a process of intrinsic targeting was adopted modelled on the Marmot Report (2010) approach of *proportionate universalism.* By designing the project to be attractive to under-represented communities, working with project partners embedded within those communities to support our access to potential participants, and by delivering in venues within those communities, recruitment was targeted towards South Asian women (Belgrave and Oadby) and women from deprived neighbourhoods (Belgrave and Braunstone).

### Aim, design and setting

In this final cycle of action research we aimed to assess the impact of the project, particularly on social capital [14] and explore the factors underpinning the development of social capital by collecting qualitative data on participant and dance tutor experiences using focus groups embedded in the existing model of the project. Thus, focus groups were conducted in the dance studio of Desi Masti dance school in Belgrave and in the church hall of All Saints Church in Braunstone.

### Characteristics of participants

Participants consented to the focus groups, but some did not provide protected characteristic data on age, ethnicity and sex. Those who did not wish to provide protected characteristics data described how this made them feel vulnerable, so no attempt was made to require this. In total 18 people took part, 10 women and one man from Braunstone, and 7 women from Belgrave. However, only 4 people from Braunstone wanted to provide information on protected characteristics.

All participants in Belgrave were Asian British Indian women aged between 59 and 79 (mean average 71). The tutor is female, in her 20 s and from an Asian Indian background; she is resident within the local neighbourhood.

Participants in Braunstone were aged between 22–83 (mean age 56.5), most were female and the majority were White British. The tutor is female, in her 20 s and White British. She does not live within the Braunstone neighbourhood.

The (lead) researcher is female, in her 40 s and of a mixed White background (Irish and British). Whilst she is a third generation migrant, like most Asian people of a similar age in Leicester, she recognises that she has experienced privileges associated with her ethnicity that remove her from many of the experiences of Asian women. Her socio-economic background is the Leicestershire coal mining community.

### Description of processes

All project partners, including participating women, felt that success would feature broad criteria like learning new things, making new friendships, and building community, simply getting active, and social and wellbeing benefits. As such, there was support for a process to measure softer outcomes and collect qualitative data through focus groups. This approach fit well with the existing model of the project, allowing us to embed the focus groups within the discussion sessions of normal delivery. Interviews were considered but the participating women preferred to share the discussion as a group. Topic guides were developed for the dance teachers and the project participants. Focus groups with project participants and dance tutors were conducted in the final summer dance and health sessions in June 2022 using the same format as for discussions; participants sat on chairs in a circle in their respective venue, dance tutors joined us, and no other people were present. Focus groups lasted 45–60 min, slightly longer than the usual discussion. It was deemed necessary by the researcher to run the focus group over two sessions in Belgrave, as many of the participants spoke English as a second language. As a result it sometimes took a little more exploration, prompting and rephrasing for the researcher to get a clear idea of the participants thoughts and feelings. Interviews with representatives of the community project partners, Aakash Odedra Dance Company and Moving Together, were conducted in July 2022 and recorded, in addition to participating in the focus groups, to allow them to fully explore their experiences.

### Description of processes of analysis

The majority of participants took part in the focus group discussions. Qualitative data was analysed using qualitative content analysis.

A full data analysis plan was determined as part of the action research process [19]. Qualitative content analysis of transcribed focus groups and interviews was undertaken.

Some themes were previously generated through the process of iterative development of the project model of engagement and the ongoing review of relevant literature embedded in the action research process. As such, the focus group and interview data presented itself more as a means of evaluating those themes than as raw data at the start of a discrete project. In the context of content analysis, the action research cycles associated with iterative development of the model of engagement incorporated theory-guided development of themes of coding and also allowed for the pilot testing of those codes [22].

As such, themes generated to describe the impacts previously observed, were applied to transcribed focus group and interview data. Recognising that the project team have not been able to identify all impacts or anticipate all themes that the focus group participants themselves considered important, new themes were identified to describe data that did not fit the existing themes; we anticipated this is likely as the process up to this point had been primarily undertaken by the researcher and the project team and was unlikely to recognise impacts or concerns that effect participants comprehensively. The process was therefore both deductive and inductive.

In qualitative content analysis the focus is on the credibility and trustworthiness of themes over their replicability and validity, acknowledging the subjective and descriptive nature of the findings [23]. This fits into Sagor’s [19] model of action research, as each cycle presented an opportunity to establish credibility and trustworthiness by referring back to existing theory and research, and incorporating new theory and research as it became relevant.

The project model also presented opportunity to embed researcher reflexivity and self-reflection in a comprehensive manner, to support our capacity to generate trustworthy and credible themes. Finlay [24] identifies five facets of reflexivity and self-reflection and we consider how four of these are embedded into the project.

1. Introspection—the ongoing process of action research embedded introspection into the project.
2. Inter-subjective reflection—regular meetings with project team members were embedded to support both relational and operational aspects of the project. These embedded opportunities to develop our understanding of the project impact across perspectives and utilising the different cultural competences of the project team members. This was fundamental to the process of developing the model of engagement and provided a source of data for the action research cycle without excessively burdening project participants.
3. Mutual collaboration—this entailed supporting project participants to increasingly contribute to the development of the model of engagement and our understanding of its impact.
4. Social critique—this describes ways in which we sought to overcome power imbalances for the purposes of facilitating open and honest communication between project participants and the researcher. Researchers have formerly used approaches like humour [24]. The use of culturally popular dance forms supported this by presenting the researcher as vulnerable and through the experiences of laughing and learning together.
5. Discursive deconstruction—this approach to reflexivity was not embedded within this project.

Initial familiarisation was conducted through the transcription process.

Subsequent coding categorised data into themes. Themes were re-examined to check they fit the data, and identified that the main themes generally fit the data well but that some data fit better into themes that overlapped those derived from the preceding work, e.g. relationship building.

The long form of GRIPP2 (Additional File 1) was used to report our involvement activities, providing more details on the contextual development of the project between 2017–2022 and a critical reflection of the project.

### Ethics

Ethical review of the research component of the project was undertaken by the Social Research Ethics Group at the Deanery of Biomedical Sciences at the University of Edinburgh. Consent was recorded on an informed consent form and informed through presentation of the project by the researcher as part of a ‘group consenting’ process in which the participant information was presented to the group and discussed prior to individual discussions and consent form completion. It is important to note that participants were free to participate in the project without taking part in the interviews or focus groups, or allowing the processing of their data (2 people declined). This process was supported by a participant information sheet. Consent from personnel representing the dance partners was also sought and recorded on an informed consent form and participant information sheet. The consent process with dance partners was conducted in a one to one discussion.

## Results

The qualitative content analysis of the focus group transcripts identified the following themes in the data. All themes are included, although some do not relate to the field of social capital, in recognition of their importance to participants.

### Feedback on the project

A considerable amount of data focussed on how participants valued and enjoyed the project, and particularly the dancing.

**Table:** 

Int: So my first question to you as a group is um how did you feel about taking part in the dancing?
Ppt: I enjoyed it
Ppt: Hmmm
Ppt: It’s got me out of the house as well because this is my day off and that yeah look forward to it (Braunstone)

**Table:** 

Int: So, tell me, how did you feel about taking part in the dancing?
Ppt: Excellent
Ppt: Very nice
Ppt: … Excellent, it was very nice, brilliant (Belgrave)

It is pleasing to note at this stage that there was no negative feedback in any of the focus groups. Whilst for most people dance was the primary factor that attracted them to the project, some participants did identify that the health focus of the project was also attractive.

**Table:** 

Int: So it was mainly the dance classes that were attractive?
Ppt: ‘No, no, health for me, definitely. (Braunstone)

Feedback identified that project personnel were pivotal in making the project appealing. Specifically, participants valued having a consistent team who are friendly and who had the relevant skills in either dance tuition, science communication or cultural competence.

**Table:** 

Int: Um, did you have any experiences where other people in the group encouraged you or made you feel stronger?
Ppt: [Tutor Name] is very encouraging
Ppt: With [Tutor Name], she’s…
Ppt: We miss her when she’s not here
Ppt: The way she smiles when she talks I think for me it helps
Ppt: Ha ha it helps when I squat!
[Laughter, raucous] (Braunstone)

**Table:** 

Int: Did you um learn anything new about health and science?
Ppt: Oh yes lots, lots of aspects made me think oh I hadn’t realized that
Ppt: I even went home and tried to regurgitate it all but I couldn’t say it in the same way as you did so
Int: Ah like share what you learned?
Ppt: Yeah
Ppt: Exactly, so it made an impact, it has made an impact (Braunstone)

**Table:** 

Int: …so anyone else have any anxieties about coming to the sessions?
Ppt: Not now because we know you and the way you treat us is really good
[Murmurs of agreement]
Ppt: You´ve got lots of patience with us
Ppt: You´re very patient with us old ladies [laughter]
Ppt: You are really lovely
Ppt: No you´re lovely, that´s why I enjoy it. [Laughter]
Ppt: You make us laugh, [make] jokes…
Ppt: You make us feel at ease (Belgrave)

The tutor from Desi Masti highlighted how the project teams’ cultural competence, and particularly effective communication with women who spoke English as a second language contrasted with how visiting clinical and academic researchers struggled with this. This highlights the need for a skilled core team to support the wider clinical and academic research team to engage effectively with under-represented communities.

**Table:** 

Int: So thinking of the discussions that we’ve had, can you think of things that have been done badly?
…
Tut:..it was one of the last two sessions, with the researchers and I think they weren’t explaining themselves to our participants very well because they didn’t understand how our participants needed to be spoken to, or how they spoke or how they understood English for example. (Tutor Interview)

### Motivation to exercise

Participants communicated that they were motivated to attend the sessions and that this helped them incorporate exercise into the lifestyle. Motivation was achieved through a number of mechanisms including accountability to a buddy, designated time and place for exercise, or intrinsic rewards associated with the project such as the social opportunity it presented or the sense of wellbeing it created. This was consistent across the Belgrave group and the Braunstone group. In the following conversation between focus group participants they discuss how the project helped them exercise:

**Table:** 

Int: So my first question to you as a group is um how did you feel about taking part in the dancing?
Ppt: I’ve found it motivated me to do exercise because when I’m at home on my own I probably do[n’t] because I’m on my own but with a group I feel more motivated to do it.’ [Short silence]
Ppt: It’s the socialising as well isn’t it? [Short silence]
Ppt: I usually come with and sometimes I probably wouldn’t have come to a class but J is coming and I’m thinking oh no I’ve gotta come because J is coming
Ppt: Encouragement
Ppt: That accountability. Someone to be like you gotta come, come on
Ppt: Notice if you’re absent or late. (Braunstone)

### Criticisms of the NHS

The focus group discussion frequently went in the direction of criticism of the NHS, with the topic persistently arising despite there being no real focus on clinical care in the topic guide questions. This suggests that difficulty accessing the NHS is of significant concern to the participating women. The underlying theme may relate to the greater challenges faced in accessing healthcare in communities with lower social capital. Sub-themes within this topic included inaccessibility, a shift in focus from prevention to sickness and even only really urgent care, inconsistent care and a sense of declining standards. A decrease in trust was specifically mentioned.

**Table:** 

Int: If you think about doctors what feelings come to your mind? Bearing in mind I won’t be showing any of them this recording
Ppt: I feel they’re unaccessible at the minute [giggle]
Ppt: I’ve forgotten what mine looks like [laughter, agreement]…
Ppt: Yeah I am wanting to see my GP for months but it’s not GP, it’s the receptionist. I hope there’s no receptionists in the room. [laughter] and it’s not with the GP it’s with them I have an issue and then I asked for the practice manager
Ppt: Ah yeah…
Int: Are doctors people we trust?
Ppt: I trust the doctors but I prefer to see them face to face, not on telephone
Ppt: Yeah
Ppt: Yeah I agree with that (Belgrave)

In the following conversation between the participants with a prompt from the interviewer they express their growing distrust of the NHS, and link it to difficulty accessing services and feeling rushed whilst using services.

**Table:** 

Ppt: Certain things that have happened I don´t trust the NHS
Int: No? [Short silence]. So it is hard to trust the NHS
Ppt: It feels like everything is going down hill
Ppt: Yeah it feels like it´s more difficult to get into services and use them, isn´t it?
Ppt: Um, the care is not there
Ppt: People need the services um [unclear] and last time it was just in and out (Belgrave)

Throughout the focus group discussion there was no positive comment on receipt of healthcare or the NHS. All discussion was negative.

### Mental wellbeing

Whilst the aim of the project was never particularly to create positive clinical benefit, it certainly achieved this in the opinion of participants most notably in the area of mental wellness. This was consistently stated as an example of the benefits of taking part in the project.

**Table:** 

Int: Er, does dancing effect your mood?
*…*
Ppt: I think it lifts you, it lifts your mood doesn’t it? Makes you feel better so if you come along feeling a bit ugh I’m not sure I can really do this today sort of 15 min in you’re thinking ah that’s better (Braunstone)

**Table:** 

Int: Do you, do you um what do you feel like you´ve achieved taking part in these sessions
…
Ppt: If um mentally, our mental health has improved especially with COVID. (Belgrave)

### Engagement in a post pandemic new normal

As identified, there was fearfulness and a sense of high emotional labour associated with participating in the project after living through the COVID-19 pandemic. This was evident at several points in the transcripts, often in reaction to discussion about the value of face to face and group based engagement, as illustrated in the following discussion between participants.

**Table:** 

Int: And how did it feel coming out of COVID and starting dance classes again?
Ppt: It´s much better
Int: Yeah?
Ppt: Personally
Int: Were you nervous at all?
Ppt: A little bit
Ppt: Yeah a little bit?…
Ppt: At the beginning
Ppt: Yeah very nervous at the beginning
Ppt: Yeah when we came the first time
Ppt: It feels like it took a lot more energy and concentration
Ppt: And even now we have to be very very very very very careful
Ppt: Yeah, yeah yeah it was important to be very careful. (Belgrave)

### Accessibility and inclusivity

A number of themes fit into the construct of accessibility and inclusivity. This meant different things to different people, although there were shared ideas about what made the project accessible to and inclusive of participants. Two key facets of accessibility for the participants were that sessions were free to join and that they were group activities where participants knew other people. In discussing whether they experienced feelings of uncertainty or anxiety attending the classes at the beginning of the project:

**Table:** 

Int: So, tell me, how did you feel about taking part in the dancing? …
Ppt: At the beginning it’s a bit scary, made me anxious, well I was anxious at the beginning. I don’t know about anybody else?
Int: How about coming to the sessions?
Ppt: No not the sessions, sorry. I’m talking about the times we performed
Ppt: The performance. Yeah
Ppt: But not the dance classes. No, because we know everybody here. (Belgrave)

**Table:** 

Int: Um, what do you think communities should do to improve health? Not individuals, but collectively communities…
Ppt: And I think our council should, cos not everybody can afford to go to these classes, and I think that the council should look into subsidising something or whatever (Belgrave)

The construct of accessibility and inclusivity included a welcoming, friendly and accepting group suggesting that the group norms were also important in creating an accessible and inclusive culture within the project. This was considered important in creating a group that embraced the diversity of the community.

**Table:** 

Int: How do you feel you get on with the other people in the group? Have you forged new friendships? [sounds of agreement]
Ppt: I think this has been a really… happy group. Very friendly
Ppt: Um, we said that when we first came. That we sort of interacted with each other didn’t we? …
Ppt: Especially since it is such a varied group with age, abilities, backgrounds like we’ve got like from 30 plus it’s such a wide range isn’t it so I think it’s so great how you all got on (Braunstone)

**Table:** 

Int: Did you feel like you got on with people in the class? Were you part of the group?
Tut: …it’s like a family setting. It feels like we’re all kind of one and when we come together, no matter what everyone’s background is… we kind of become one when we come together for the class. (Tutor Interview)

**Table:** 

Int: So, as a very diverse group do you do you feel like you belong? [Sounds of agreement]…
Ppt: I think me here with my disability I feel very comfortable because I feel I’m accepted as I am and everyone accepts me, and I can just move forward so I’m quite comfortable in this group. (Braunstone)

The project staff were identified as key to creating initial reassurance and a welcoming environment to overcome any feelings of anxiety or uncertainty that people had around joining the sessions. Preceding the discussion about qualities of project staff (provided in the project feedback section) a participant discussed her fears that the project staff would not be welcoming.

**Table:** 

Int: Um was there anything that you were um worried about when you were preparing to come to the dance classes? Think right back to when we first started. Was there any sense of anxiety about coming to these sessions?
Ppt: …Well, I thought, well I didn’t know the teacher at that point and I thought if she shouts at me I won’t come back. (Braunstone)

A participant affected by visual impairment was specifically concerned about accessibility. Following on from the discussion above she added:

**Table:** 

Ppt: I think I felt a bit nervous because I’m blind. I thought I wouldn’t be able to manage but you know when [Tutor Name] was doing the moves but I think she helps me by showing me an action and standing in front also helps me be able to do some of the moves. So I feel quite OK now. (Braunstone)	

These comments also highlight the psychological barriers that may prevent people from participating in community engagement projects, such as concern that project leaders may be mean or unfriendly, or unable to accommodate the participants needs.

### Building social capital

The project in and of itself was valued by participants, as illustrated by the positive comments in a preceding section on the theme: feedback on the project. Furthermore, the project offered an opportunity for participants to access a space where they could invest in themselves and their health.

**Table:** 

Int: Do you think that the actual activity of dancing made it easier to get to know people? [agreement] And can you tell me why? Why dancing?…
Ppt: And it´s also um stress free um you know there’s stress at home you’re stressing and then when you come here our stress is all finished. (Belgrave)

Many responses focussed on the importance of relationship building. This articulated ideas around overall team building, creating friendships with each other and building relationships between project partners and project participants.

**Table:** 

Int: How did you feel that you got on with people in the class?
Ppt: Very friendly
Ppt: Lots of friends
Ppt: Like people I didn´t know I made friends with them now. (Belgrave)

The importance of project staff joining in as a means to create a level playing field and dismantle any perceived differences in social position was emphasised.

**Table:** 

Int: Um, so in the course of the sessions you’ve had an opportunity to speak predominantly to me and occasionally to other researchers. What was it like meeting people like me away from a hospital or a lab and in the real world? Was it different?
Ppt: It was very interesting
Ppt: The fact that you joined in as well
Int: Tell me about what difference joining in made. I’m interested in that
Ppt: Cos we’re together
Ppt: You weren’t just separate
Ppt: On the side
Ppt: One of us
Ppt: You weren’t observing and then saying now I’m going to talk to you about this
Ppt: You were part of the group
Ppt: It’s probably made you more approachable, more relatable, being part of the group and then…
Ppt: It’s a bit of a level playing ground. (Braunstone)

Around the theme of building a community around the project, a team that included the women as project partners, and shared cultural references were considered important in the Belgrave group (Asian women).

**Table:** 

Int: Do you think that the actual activity of dancing made it easier to get to know people? [agreement] And can you tell me why? Why dancing?
Ppt: Because when we dance it´s the best chance to um we know the songs, so we can join in easy. (Belgrave)

**Table:** 

Int: Are there particular ways you think would be effective at getting people, especially people from the community that we’ve worked with, interested in health and science?
Tut: I think by by bringing activities like this where they get to do something physical or something that they’re interested in and we can say that with this you’ll also get the chance to have a chat about anything that you need because I think when we approach the community and tell them that we can talk about health they may not want to just come for a health talk because that sometimes scares them away because they don’t fully understand it but I think when there’s something they relate to or that they’ve got a connection with first they can come to that and have the health talk added on to it cos it helps them relate more
Int: It certainly helped us get people interested that’s for sure
Tut: It did, it did, because our participants for sure they probably wouldn’t have sat and had a circle discussion on health if it wasn’t for a setting that they are comfortable with. (Tutor Interview)

Dance was considered to be an effective way to create feelings of togetherness through shared vulnerability, such as doing sexy dance moves or sharing the experience of learning and making mistakes together, and through the communal nature of the activity facilitating things like eye contact and spatial awareness of others around us.

**Table:** 

Int: Um, did the dancing make it easier to get to know people? Not just the being here, so not just that there is an activity, but the actual specific activity of dancing? Together
*…*
Ppt: If it had been a floor based exercise routine then we wouldn’t have had all the that eye contact that you instinctively have with people …
Ppt: ‘I think if we have a laugh too you kind of look at each other like here [Tutor Name] goes again or you can like have your own little know what I mean little glances or oh I just messed up. I think it’s like, it’s a way to acknowledge each other with your facial expressions because I see it all.. and it’s beautiful.’ (Braunstone)

**Table:** 

Int: …so anyone else have any anxieties about coming to the sessions?
Ppt: And you know like J some of the steps you showed us I used to that sometimes they were a bit rude but when you’re doing them together it doesn’t feel like you don’t feel like [murmurs of agreement and laughter expressing that the ladies didn’t feel embarrassed by sexy dance moves]. (Belgrave)

**Table:** 

Int: And did dance help you to share how you feel with people in the group?
Tut: …everytime I danced with them it was less of me dancing at them and it was more us dancing together and sharing our own ideas and being able to be free and be open in the class. (Tutor Interview)

Participants recognised the role of trust building in the project and readily related this to their feelings of low trust in the healthcare and health sciences. Low and deteriorating levels of trust in healthcare are described in a preceding theme considering criticisms of the NHS. There was also frustration related to not being believed or trusted by healthcare professionals, which may particularly relate to the sex of the project participants.

**Table:** 

Int: So if you’re looking for advice to keep healthy, where or to who might you go?	
[Discussion about various sources leading onto GPs]	
Ppt: You have to tell them what you need and then they don’t believe you. (Braunstone)	

More specifically concerned with science there was a level of distrust that was explicitly exacerbated by the COVID-19 pandemic. Discussing scientists:

**Table:** 

Int: Do you feel like you trust them?’	
Ppt: No. [laughter]	
Ppt: No I think that… [loud laughter]… I trust them but I think they should do more research because I know they tried to like give us the jab, you know, as safe but I still feel that more research needs to be done. (Braunstone)	

There was a tendency to frame research as a necessary evil. Leading on from the above conversation:

**Table:** 

Ppt: I don’t think we can progress without it. (Braunstone)	

However, through the project the opportunity to discuss the nature of science and research, and how more information can change what we believe to be best practice, as well as the opportunity to get to know a representative of the world of research (the first author) specifically as a fallible human being, trust was built.

**Table:** 

Int: … you’ve asked me a question today and I didn’t know the answer. Does that change how you’d feel about advice that I gave you because I didn’t know everything? Or would you still feel confident around that advice? …	
Ppt: I think it makes it more truthful because you’re being honest, I don’t know that makes us believe what you’re saying more cos you’re like you know what I mean being honest about what you do and don’t know and then you value your knowledge and what you are saying	
Int: It sounds like that’s alluding to trust? [sounds of agreement]	
Ppt: I agree. I would be very confident with that you tell me because I know you’re studying and you’re doing research and things like that and erm and you make sound advice and some others may give something else isn’t it? So you can take it or leave it so. So I’m quite confident and I trust you. [sounds of agreement]	
Ppt: And even like earlier when we were talking about digestion and you were like I know someone who is doing a paper on this and it’s actually the opposite like you don’t say like this is fact you say like this is some information I know take it or leave it type thing. (Braunstone)	

Feedback highlighted how participation in health-related public engagement made participants more conscious of health.

**Table:** 

Ppt: Did these classes inspire you to think about your health?	
*…*	
Ppt: I think it’s interesting how this activity, um, helps you think about health more and your mental health more. How it impacts on that. …Not just thinking about health generally but.. the activity that you’re doing makes you think about your health. (Belgrave)	

A reflective approach in discussions was considered to play a role in maximising potential impact for participants. Leading on from the same conversation:

**Table:** 

Ppt: And I think that the chats that we do always encourage people to be reflective of themselves as well, whether people say things in the group or not I can see everyone thinking about the questions that you ask and it gives everyone a chance to just reflect on how they found it and stuff which I think is nice. (Belgrave)	

### Empowerment

The discussions highlighted ways in which the women felt they took ownership and responsibility in the project. These are conceptualised as empowerment in the context of the shift in sense of responsibility and ownership of the project throughout the process of its development and delivery between 2017–2022.

**Table:** 

Int: So, as a very diverse group do you do you feel like you belong? [agreement]	
Ppt: This is MY dance group [laughter and agreement] (Braunstone)	

Discussion also referenced ways in which the project staff did not try to control interactions or dictate session content. It is likely this facilitated the process of empowering participating women to take ownership and responsibility within the project.

**Table:** 

Int: Did you uh find yourself talking to me or to the other visiting speakers at all about stuff that wasn’t related to health like social chat?	
*…*	
Ppt: Yeah we can easily go off track and talk about whatever and bring it back to dance, but like it’s nice that Becky [first author] like allows that. I think that then adds to the social building of like community. (Braunstone)	

**Table:** 

Int: So were there things that worked?	
Ppt: You’ve always tried to give each person a chance to give their views and that’s kind of helped them not be scared to talk. (Tutor Interview)	

Project participants felt there was a balance between project staff and participants that was important to building their role within the project as empowered project partners. This was specifically related to the researcher joining in the activity.

**Table:** 

Int: Tell me about what difference [the researcher] joining in made. I’m interested in that	
*…*	
Ppt: You ask us questions and we can ask you questions. (Braunstone)	

## Discussion

The results support the claims of O’Mara-Eves [1] that a safe and accessible project is necessary for effective community engagement and that this includes factors like cultural familiarity. The focus group discussions suggest that there are several facets to the constructs of safe and accessible when applied to community engagement with under-represented and deprived communities, and possibly more generally. The focus groups expanded this to emphasise the importance of cultural relevance, a sense of belonging, norms and leadership that made the group welcoming and friendly, and flexibility that allowed us to be inclusive of participants with a range of different abilities. The results demonstrate that the model of community engagement/public involvement supported bonding opportunities which in turn supported the development of social capital [15]. The importance of inclusion of the researcher (and visiting clinical and academic researchers from the research centres undertaking public involvement) in the bonding activity suggests that inclusion of outsiders in bonding can support bridging. This further emphasises that bonding and bridging are not mutually exclusive constructs [15]. Thus whilst we recognise that ability to get involved in research through public involvement is a function of social capital [25], creating culturally accessible inclusive models of involvement can support development of social capital.

The results also reflected the domains identified by Sung [7] as determinants of effective community engagement (flexibility, a sense of belonging, commitment, communication, being genuine, relevance, sustainability). Participants identified the offer of dance was relevant and inviting, but also that the health component was attractive, though there was considerably stronger enthusiasm for dance, supporting the idea that the arts are an effective means to get people engaged with science. Participants particularly valued the effect of the sessions on their mood, both through dance and the social component of health discussions, and through simply allocating a space dedicated to self-care which Belgrave (Asian) participants particularly considered as distinct from everyday life. This highlights the ongoing relevance of inclusion of mental wellbeing in engagement focused on physical health as an important and desirable outcome for participants; it highlights the desirability of holism in our approach. The general positive feedback concerning the project also implies that the project is relevant to participants. Participants focused extensively on the sense of belonging engendered by the project. It mattered to participants that familiar faces would be present, and particularly so for Asian participants when they were first getting involved. The activity of dance was felt to engender this sense of belonging, as were the group norms and leadership that created a culture of friendliness and acceptance, even amongst those who felt different, for example, because of disability. The group identified the role of project personnel in creating the group culture, noting that it was important that I had been involved in the activities and not separated myself from them and set myself up in a position of authority. This in turn relates to the perception that project personnel, and particularly the lead researcher, seemed genuine both socially in terms of being open and friendly and ‘having a laugh’ and professionally in terms of being open and honest about what they knew and did not, and how science doesn’t reflect an absolute truth. There was some discussion reflecting the value of flexibility in creating accessibility, inclusivity and a safe environment, for example around restarting the project after COVID restrictions were lifted, but also around creating a group to which diverse people with diverse levels of ability felt they belonged; for example the adaptations made to include a participant with visual impairment or participants who were older. This was also considered an important component of the effective facilitation of health discussions. Effective communication was related to skill, for example the ability to communicate complex scientific ideas well, cultural competence such as being able to communicate well with people who spoke English as a second language, but also as a component of the leadership creating a welcoming, friendly, inclusive and accessible group. There was less of a focus on the role of commitment and sustainability though discussion about the researcher taking part may be interpreted to allude to commitment. There was some discussion around the role of friendships and motivation to exercise and attend which may provide some insight into how the friendly group setting supported ongoing engagement.

Focus group results provided some evidence to support the claim that community engagement can lead to empowerment [16] with the project participants talking about how they felt ownership and how they had an equal role with the researcher and a relationship of ‘give and take’. It has been suggested that empowerment is a factor in building social capital [17]. Project participants certainly expressed both a sense of empowerment and gave examples of growth of social capital with a primary focus on relationship building. This included friendships which provided various sources of resilience including motivation, accountability, encouragement, fun, positive affect and social benefits. It also included the development of relationships with the project personnel, which underpinned effective engagement and related health benefits. There were also examples in which participants specifically alluded to a sense of ownership, equality and empowerment through their participation. Dance, as a bonding activity that included outsiders therefore also functioned as a bridging activity. The results show that engagement led to empowerment [16] and that this may underpin development of social capital [17] but specifically where the community engagement moves towards the practice of coproduction, by foregrounding the principles of flexibility and relevance which underpin the effectiveness of community engagement [7].

Cultural attitudes create low levels of self-care particularly amongst South Asian women, which may manifest in small amounts of leisure time, low levels of physical activity and poor diet [26]. Reflections in the focus group amongst the Belgrave (Asian) participants that a stress-free and welcoming space dedicated to self-care was valuable, relevant and in contrast to their homelife, and their willingness to make use of such spaces may offer an opportunity to address generally low levels of physical activity evidenced in previous research amongst Asian women in the UK [27]. As the project was effective in engaging with and building collaboration with women from South Asian and White British deprived communities in Leicester, this may present an opportunity to support the shift towards self-care and away from cultural authoritarian perspectives, that tend to silo healthcare as the domain of experts as opposed a function of lifestyle [28–31].

Participants described how the project offers health benefits at multiple levels. The project provided an opportunity for healthy physical activity directly to participants. The health engagement component offered information and the opportunity to reflect on health behaviours. The project itself created social capital; it built relationships and friendships, and facilitated access to information in a health positive group culture. Within the results there is evidence of both bonding and bridging, with the participants referencing how the activity was an opportunity to meet people and form friendships within a culturally safe space (bonding) and also learn about a different culture (academic research and science) and establish trust with people from that culture (bridging) through their inclusion in the bonding activity.

This growth in social capital is itself a resource that has potential to offer health benefits and reduce health inequalities [3, 18]. In light of the lower levels of health literacy and English literacy amongst migrant populations [4–6] the role of the project in providing access to well communicated information is likely to be valuable. Indeed, the participants highlighted how this has occurred in reference to mental wellbeing which can be applied to these three levels of impact.

1. We offered a space that provided improvement in wellbeing through dance
2. We provided information on mental wellbeing and discussed how to look after our mental health, notably during the COVID pandemic
3. The friendships developed support mental wellbeing beyond the project

A considerable focus of discussions was around how dance was effective at underpinning the process of building relationships and creating a welcoming group. Participants referenced the ways in which dance created social interaction and engagement, consistent with previous research [8, 9]. They reflected on the role of eye-contact, shared cultural points of reference, and particularly focused on vulnerability, for example, making mistakes or doing sexy dance moves that built humour and occasionally raucousness that engendered the sense of shared experience. The focus group discussions also highlighted how the cultural relevance and familiarity of the type of dance and music was important to the Belgrave (Asian) participants. Previous research has also identified how dance as an activity offers more than just physical activity [10, 11] and the reflections on mental wellbeing, building social capital and motivation to change health behaviours reflect that incorporating dance in these sessions has indeed had wide ranging benefits. Utilisation of dance ultimately offered far more than simply the power to obtain an audience [13], but also reduced social isolation [8, 9] and was a tool underpinning bonding and bridging.

The focus of the results on the role of communication and leadership from project personnel provide some limited further insight into the role of gatekeepers. The importance of providing a culturally safe space was recognised [32], and this can be seen as a function of development of cultural competence supported through effective partnership working.

### Implications for policy

The project provides insight into a model of community engagement and public involvement and engagement that demonstrates how health research can work effectively with minoritised communities and with meaningful impacts within those communities. However, the primary implications for the practice of public involvement and community engagement are that redistributive approaches to social justice (getting people from under-represented communities to participate in public involvement or community engagement we already do that aligns to dominant cultural norms) are insufficient and we need to focus on relational social justice (new ways of doing public involvement or community engagement that aligns with under-represented cultural norms). This is consistent with findings in science communication [33]. Delivering against the NIHR strategic objective of greater inclusion in public involvement cannot be seen as a simple process of removing barriers, but demands a restructuring of the very concept of engagement and involvement to make it meaningful beyond the norms of the dominant culture.

A significant and unsurprising implication of this reflection, and the project’s support for Sung et als (2013) criteria is that public involvement and community engagement funding is insufficient and insecure. It does not currently offer sufficient investment in the management capacities nor the systemic restructuring of practice required to move towards relational justice.

The model of engagement used also suggests there are implications for the NIHR guidelines (2023) on how we should reward and recognise people who participate in public involvement. Where the emphasis of the guidance is on making individual payments to people who participate in public involvement, the Dance and Health Project suggests that community investment may be as highly valued as a gift voucher or payment and, importantly, have the potential to impact health inequalities and social capital within the target communities in ways that individual payment cannot. As such there is a need to consider how the NIHR guidance can be adjusted to allow for community investment models of reward and recognition and in such a way as will not be exploited to avoid individual payments where these may be more appropriate.

### Study limitations

The action research approach intrinsically supported the development of empathy and compassion [34] as the project evolved from self-reflective approaches that predominantly sat with the researcher, to participatory action research involving the project partners and subsequently the participating women [35], and finally to a more politically driven emancipatory approach [36] where we started to explore the implications for our findings on approaches to public involvement in the context of social justice.

We recognise that action research has limitations, including its openness to bias from the researcher and her collaborators, and its limited generalisability. By actively linking the process to existing research we try to ensure that we remain as objective as possible. Furthermore, the role of the researcher in project delivery has both advantages and disadvantages. Whilst the development of a strong relationship with participating women over the preceding months established openness and trust it may discourage negative feedback.

Inclusion of the impact assessment component of the project provided an opportunity to consider project impact in more depth using content analysis. The credibility of the themes identified is underpinned by embedding reflexive practice and reflection in the very processes of project management. This underpinned development of themes through not only introspection, but also inter-subjective reflection, mutual collaboration and social critique [24].

Therefore, whilst content analysis may be deemed subjective as an approach, we can assert that the project embedded a comprehensive approach to the development of our understanding of the project and its impacts that supports the credibility and trustworthiness of the themes identified.

### Future studies

The results of this project suggest that assessment of impacts of public involvement and community engagement should incorporate a broad focus with an emphasis on development of social capital and impact on health inequalities. Future studies on the applicability of the model of engagement and focus of impact assessment in diverse communities would be valuable.

### Supplementary Information

Supplementary Material 1.

## Data Availability

The datasets used and/or analysed during the current study are available from the corresponding author on reasonable request.
