# Supplementary material for: Enhancing community engagement, public involvement, and social capital through researchers’ participation in community dance projects: unexpected outcomes in underserved communities
Source: Res Involv Engagem. 2024 Aug 2;10:79. doi: 10.1186/s40900-024-00616-9 (PMC11297768; doi:10.1186/s40900-024-00616-9)
Supplement: Supplementary file 1 — Supplementary Material 1. [file 40900_2024_616_MOESM1_ESM.docx]

| **Section and topic** | **Item** | **Reported on page No** |
| --- | --- | --- |
| Section 1: Abstract of paper | | |
| 1a: Aim | The study aimed to assess if a community engagement intervention was effective at building social capital. | 6 |
| 1b: Methods | Community participants were project partners in the iterative development of the engagement model (Dance and Health) between 2017 and 2021. The conceptualisation of project success and associated outcomes were jointly decided by all project partners including participants.  The impact of project delivery in 2021/22 was explored using focus groups and interviews. | 7 |
| 1c: Results | The focus groups and interviews found that Dance and Health was a viable model of community engagement offering an opportunity to deliver public involvement in a culturally accessible format | 20 |
| 1d:Conclusions | The culturally accessible format of Dance and Health supports empowerment and represents a form of relational social justice in that it makes public involvement accessible and meaningful to those experiencing inter-sectional exclusion. There is evidence to support the success of the project in developing social capital through bonding and bridging. | 20-24 |
| 1e: Keywords | community engagement; social capital; public involvement; underserved communities; ethnic minority; dance | 2 |
| Section 2: Background to paper | | |
| 2a: Definition | The NIHR (INVOLVE) definition of public involvement was used. |  |
| 2b: Theoretical underpinnings | The public involvement was underpinned by good practice guidance from the NIHR, the existing research of O’Mara Eves et al (1), Sung (4), Ocloo and Matthews (7), Bordieu’s model of social capital and participatory approaches to research. | 4-5 |
| 2c: Concepts and theory development | Bordieu’s model of social capital was fundamental in supporting understanding of the wide ranging impact of the Dance and Health project. | 4-5 |
| Section 3: Aims of paper | | |
| 3: Aim | The study aimed to assess if a community engagement intervention was effective at building social capital. | 6 |
| Section 4: Methods of paper | | |
| 4a: Design | Community participants were project partners in the iterative development of the engagement model (Dance and Health) between 2017 and 2021. The conceptualisation of project success and associated outcomes were jointly decided by all project partners including participants.  The impact of project delivery in 2021/22 was explored using focus groups and interviews. | 7 |
| 4b: People involved | All participants in Belgrave were Asian British Indian women aged between 59 and 79 (mean average 71). Participants in Braunstone were aged between 22-83 (mean age 56.5), all were female and the majority were White British. | 6 |
| 4c: Stages of involvement | Community participants were project partners in the iterative development of the engagement model (Dance and Health) between 2017 and 2021. The conceptualisation of project success and associated outcomes were jointly decided by all project partners including participants.  The impact of project delivery in 2021/22 was explored using focus groups and interviews. At this stage, project participants were also focus group participants. | 7 |
| 4d: Level or nature of involvement | The project and associated research were coproduced. |  |
| Section 5: Capture or measurement of PPI impact | | |
| 5a: Qualitative evidence of impact | The impact of project delivery in 2021/22 was explored using focus groups and interviews. At this stage, project participants were also focus group participants. | 7 |
| 5b: Quantitative evidence of impact | NA |  |
| 5c: Robustness of measure | The project, Dance and Health, represents the culmination of action research between 2017 and 2021. The manuscript focusses on the deeper qualitative assessment of the process of involvement and engagement and it’s impact through focus groups and interviews. Whilst this provides rich data on the project it is difficult to generalise beyond the communities involved and the region of Leicester. However, earlier in the project there were pilots to explore generalisability of findings to other communities that will be reported separately. However, in working in two of the most deprived and excluded communities in the UK, the successes of the project still yield important insights for public involvement in the wider UK context. |  |
| Section 6: Economic assessment | | |
| 6: Economic assessment | This project did not aim at exploring the economic facets of public involvement however as a model of community investment it does have potential implications for the NIHR reward and recognition policy. |  |
| Section 7: Study results | | |
| 7a: Outcomes of PPI | NA |  |
| 7b: Impacts of PPI | NA |  |
| 7c: Context of PPI | NA |  |
| 7d: Process of PPI | NA |  |
| 7ei: Theory development | NA |  |
| 7eii: Theory development | NA |  |
| 7f: Measurement | NA |  |
| 7 g: Economic assessment | Dance and Health proposes a model of community investment over individual investment which is potentially at odds with Reward and Recognition policy. |  |
| Section 8: Discussion and conclusions | | |
| 8a: Outcomes | The project as an example of community situated public involvement produced wide raging outcomes including successful engagement with under-represented communities, co-designed outcomes that described what success of the project would look like and, through the formative years of the project (2017-2021) a model of involvement arrived at through participatory action research. | 20-24 |
| 8b: Impacts | The project yielded good practice implications for working with inter-sectionally excluded groups, evidence of growth in social capital, the beginnings of an understanding of the role of relational justice in public involvement and engagement and an emphasis on the role of community engagement standards in the context of social capital development. | 20-24 |
| 8c: Definition | The NIHR (INVOLVE) definition of public involvement was used. |  |
| 8d: Theoretical underpinnings | The project yielded good practice implications for working with inter-sectionally excluded groups, evidence of growth in social capital, the beginnings of an understanding of the role of relational justice in public involvement and engagement and an emphasis on the role of community engagement standards in the context of social capital development. In respect of relational justice, the project has implications for normalised models of public involvement in research and policies like Reward and Recognition. | 20-24 |
| 8e: Context | Throughout the project, the primary challenge pertained to short term funding arrangements associated engagement. This undermined the delivery of sustainability as a primary driver of good community engagement identified by Sung (4). |  |
| 8f: Process | Foregrounding the principles of action research, and a commitment to levelling the playing field and active participation were important enabler. |  |
| 8 g: Measurement and capture of PPI impact | The impact of project delivery in 2021/22 was explored using focus groups and interviews. At this stage, project participants were also focus group participants. | 7 |
| 8 h: Economic assessment | Dance and Health proposes a model of community investment over individual investment which is potentially at odds with Reward and Recognition policy. |  |
| 8i: Reflections/critical perspective | The project, Dance and Health, represents the culmination of action research between 2017 and 2021. The manuscript focusses on the deeper qualitative assessment of the process of involvement and engagement and its impact through focus groups and interviews. Whilst this provides rich data on the project it is difficult to generalise beyond the communities involved and the region of Leicester. However, earlier in the project there were pilots to explore generalisability of findings to other communities that will be reported separately. However, in working in two of the most deprived and excluded communities in the UK, the successes of the project still yield important insights for public involvement in the wider UK context. A second focus group was required in Belgrave for participants who speak English as a second language; it sometimes took a little longer to explore complex ideas. |  |
